# ^1^H, ^13^C and ^15^N resonance assignments for the acetyltransferase domain of the kinetoplastid kinetochore protein KKT23 from *Trypanosoma brucei*

**DOI:** 10.1007/s12104-025-10235-4

**Published:** 2025-05-02

**Authors:** Patryk Ludzia, Charlotte Nugent, Bungo Akiyoshi, Christina Redfield

**Affiliations:** 1 Department of Biochemistry, University of Oxford, Oxford, OX1 3QU UK; 2 Department of Biochemistry, University of Cambridge, Cambridge, CB2 1GA UK; 3 School of Biological Sciences, Institute of Cell Biology, University of Edinburgh, Edinburgh, EH9 3BF UK

**Keywords:** KKT23, Kinetoplastid, Kinetochore, Acetyltransferase, GNAT, GCN5, Acetyl coenzyme A, Trypanosomes, NMR resonance assignments

## Abstract

KKT23 is a kinetoplastid-specific kinetochore protein that has a C-terminal GCN5-related histone acetyltransferase domain that acetylates the C-terminal tail of histone H2A. Here, we present the ^1^H, ^13^C and ^15^N resonance assignments for the C-terminal region of KKT23 (KKT23^125–348^) from *Trypanosoma brucei* in complex with known cofactors for acetyltransferases, acetyl coenzyme A and coenzyme A. These assignments provide the starting point for detailed investigation of the structure, dynamics and interactions of KKT23 in solution.

**Supplementary Information:**

The online version contains supplementary material available at 10.1007/s12104-025-10235-4.

## Biological context

During chromosome segregation, cells must accurately replicate and distribute their genetic material. Failure to do so can result in genetic abnormalities like cancer or cell death (Mcintosh 2016). A crucial structure in this process is the kinetochore, a multiprotein complex that links chromosomal DNA to spindle microtubules (Brinkley and Stubblefield 1966; Cheeseman 2014). Many kinetochore components are widely conserved across eukaryotes, particularly CENP-A, which binds DNA, and Ndc80, which forms contacts with microtubules. (Meraldi et al. 2006; Cheeseman and Desai 2008; Santaguida and Musacchio 2009; Biggins 2013; Van Hooff et al. 2017).

Interestingly, conventional kinetochore proteins have not been identified in the genomes of kinetoplastids, an evolutionarily divergent group of unicellular flagellated eukaryotes that includes parasitic species like *Trypanosoma brucei, Trypanosoma cruzi,* and *Leishmania* (Berriman et al. 2005). Instead, more than 30 unique kinetochore proteins (KKT1–25 and KKIP1–12) have been discovered in *Trypanosoma brucei.* These proteins share little to no sequence similarity with the conventional kinetochore proteins found in other eukaryotes (Akiyoshi and Gull 2014; Nerusheva and Akiyoshi 2016; D’archivio and Wickstead 2017; Nerusheva et al. 2019; Brusini et al. 2021).

In previous studies, we identified and characterised a kinetoplastid-specific kinetochore protein, KKT23 (Ludzia et al. 2025; Nerusheva et al. 2019). Using X-ray crystallography and NMR spectroscopy, we found that KKT23 contains a C-terminal acetyltransferase domain, which is structurally similar to GCN5 histone acetyltransferase and that KKT23 can acetylate the C-terminal tail of histone H2A within *T. brucei* nucleosomes (Ludzia et al. 2025). Here, we present ^1^H, ^13^C and ^15^N resonance assignments for the KKT23 GNAT domain (KKT23^125–348^) from *Trypanosoma brucei* in complex with acetyl coenzyme A (acetyl-CoA) and coenzyme A (CoA)*.* These NMR assignments form the basis for future in-depth studies on the structure, dynamics and interactions of KKT23 in solution.

## Methods and experiments

### Protein expression and purification

The DNA fragment encoding the KKT23^125–348^ protein characterised in this study was amplified from *Trypanosoma brucei* genomic DNA and cloned into the pRSFDuet-1 expression vector using NEBuilder Assembly (New England Biolabs). The *E. coli* BL21(DE3) cells were transformed with ~ 100 ng of plasmid DNA and were inoculated into 40 ml of 2xTY media containing 50 µg/ml kanamycin. Cells were grown at 37 °C overnight. The next day, cells were spun down at 3,400 g for 10 min and resuspended in 40 ml of M9 minimal medium containing 50 µg/ml kanamycin, 1 g/L ^15^NH_4_Cl and 4 g/L [^13^C]-*D*-glucose (Cambridge Isotope Laboratories) as the sole nitrogen and carbon sources. Next, the resuspended culture was inoculated into 1L of M9 minimal medium supplemented with 1 g/L ^15^NH_4_Cl, 4 g/L [^13^C]-*D*-glucose and 50 µg/ml kanamycin. Cells were grown at 37 °C to an OD_600_ of 0.9–1.0 and the protein expression was induced using 0.4 mM IPTG. The protein expression was continued overnight at 16 °C with shaking (200 rpm). A total of 6.5 L and 5 L of bacterial culture were grown to purify ^15^N- KKT23^125–348^ and ^13^C, ^15^N- KKT23^125–348^, respectively. Purification of the GNAT domain using the following protocol results in ~ 1.7 mg of pure protein per 1 L of bacterial culture.

Cells were spun down at 3,400 g at 4 °C and resuspended in lysis buffer (50 mM sodium phosphate pH 7.5, 500 mM NaCl, and 10% glycerol) supplemented with protease inhibitors (20 μg/ml leupeptin, 20 μg/ml pepstatin, 20 μg/ml E-64, 0.4 mM PMSF), benzonase nuclease (500 U/1L culture) and 0.5 mM TCEP. All subsequent extraction steps were performed at 4 °C. Cell lysis was facilitated by mechanical cell disruption (French press, 1 passage at 20,000 psi). Lysed cells were spun at 48,000 g for 30 min and the supernatant was loaded on a gravity column with TALON beads (Takara Clontech) pre-equilibrated in lysis buffer. After loading, the beads were washed extensively with lysis buffer, and proteins were eluted with elution buffer (50 mM sodium phosphate pH 7.5, 500 mM NaCl, 10% glycerol, 250 mM imidazole, 0.5 mM TCEP). To cleave the His-tag, samples were incubated with TEV protease in 1:50 w/w ratio overnight while being buffer-exchanged into 25 mM sodium phosphate, 250 mM NaCl, 5% glycerol, 5 mM imidazole, and 0.5 mM TCEP by dialysis. Note that G123 and S124 at the protein N-terminus are the result of the TEV cleavage and they precede the first residue of the GNAT domain (Q125). Next, the sample was further purified using ion exchange and size exclusion chromatography. To promote binding of protein to the ion exchange column, the sample was diluted with buffer A (25 mM HEPES pH 7.5 and 0.5 mM TCEP) to achieve the final NaCl concentration of 50 mM. Ion exchange chromatography was performed using a 6 ml RESOURCE S column (GE Healthcare) pre-equilibrated with 5% of buffer B (25 mM HEPES pH 7.5, 1 M NaCl and 0.5 mM TCEP). The protein was eluted with a linear gradient from 5 to 100% of buffer B, concentrated using 10-kD MW Amicon concentrators (Millipore), and loaded on Superdex 75 16/60 (GE Healthcare) column to further purify and buffer exchange into 50 mM sodium phosphate pH 7.0, 150 mM NaCl with 0.5 mM TCEP. Fractions containing KKT23 were pooled, concentrated to 11 mg/ml using a 10-kD MW Amicon concentrator (Millipore), and flash-frozen in liquid nitrogen for − 70 °C storage.

### NMR spectroscopy

^15^N- or ^13^C/^15^N-labelled samples of KKT23^125–348^ were used for resonance assignment in the presence of acetyl coenzyme A (acetyl-CoA) or coenzyme A (CoA) using standard triple-resonance protocols (Redfield 2015). Assignments were carried out for 435 μM protein with either 500 μM acetyl-CoA or 500 μM CoA in a 50 mM sodium phosphate buffer at pH 7.0 with 150 mM NaCl, 0.5 mM TCEP and 5% D_2_O. NMR experiments were carried out at 20 °C using 750 MHz and 950 MHz spectrometers equipped with Oxford Instruments Company magnets, Bruker Avance III HD consoles and 5 mm TCI CryoProbes.

Initially, resonance assignments for KKT23^125–348^ in the presence of acetyl-CoA were obtained using 2D ^1^H-^13^C HSQC and ^1^H-^15^N BEST-TROSY (Lescop et al. 2007; Schulte-Herbruggen and Sorensen 2000) experiments and 3D experiments including ^15^N-edited NOESY-HSQC, ^15^N-edited TOCSY-HSQC, H(CCCO)NH, (H)CC(CO)NH, HCCH-TOCSY, CCH-TOCSY, CCH-COSY, HNHA and BEST-TROSY versions (Lescop et al. 2007; Schulte-Herbruggen and Sorensen 2000) of HNCA, HN(CO)CACB, HNCO and HN(CA)CO. A more limited set of resonance assignments for KKT23^125–348^ in the presence of CoA was obtained using 2D ^1^H-^13^C HSQC and ^1^H-^15^N BEST-TROSY experiments and 3D experiments including ^15^N-edited NOESY-HSQC and BEST-TROSY versions of HNCA, HN(CO)CACB and HNCO. All 3D NMR data were collected with 25% non-uniform sampling in the two indirect dimensions using standard Bruker sampling schedules. 2D NMR data were processed using NMRPipe (Delaglio et al. 1995) and 3D NUS data were processed with the hmsIST software (Hyberts et al. 2012) and NMRPipe. Spectra were analysed and assignments recorded using CcpNmr Analysis version 2.5 (Vranken et al. 2005). ^1^H and ^13^C chemical shifts were referenced using DSS and ^15^N chemical shifts were referenced indirectly. Details of the specific experiments and sample conditions can be found in the BMRB deposition files.

Residual dipolar couplings (RDCs) were measured for KKT23^125–348^ in the presence of acetyl-CoA (twofold excess). Isotropic ^1^H^N^-^15^N splittings were measured first for a 350 μM sample of ^15^N-labelled KKT23^125–348^ with 700 μM acetyl-CoA alignment was achieved using C12E6/*n*-hexanol liquid crystals prepared as described previously (Rückert and Otting 2000). Briefly, a 15% C12E6/*n-* hexanol stock solution was prepared in HEPES buffer (25 mM HEPES, 150 mM NaCl, pH 7.15). 100 μl of this stock solution was added to 200 μl of the 350 μM ^15^N-labelled KKT23^125–348^ to achieve a final concentration of 5% C12E6/*n-*hexanol. ^1^H^N^-^15^N splittings were measured using BEST-TROSY and semi-BEST-TROSY experiments at 20 °C (Lescop et al. 2007; Schulte-Herbruggen and Sorensen 2000). RDCs were measured as the difference between the splitting observed in the isotropic and aligned data sets.

RDC values were measured for 153 of the 216 non-proline residues in KKT23^125–348^; RDC values for 63 residues could not be measured due to peak overlap. The principal components and orientation of the molecular alignment tensor were fitted to minimise the *χ*^2^ between the experimental and calculated RDCs using the KKT23^125–348^ X-ray coordinates (PDB:9EVQ) to which ^1^H had been added using X-PLOR version 3.8 (Brünger 1992). There are two molecules in the unit cell in the X-ray structure; molecule A was selected for analysis because it contains electron density for more residues. Five residues for which electron density is missing were removed from the fitting procedure. In addition, eight residues with {^1^H}-^15^N heteronuclear NOE values of less than 0.6, indicative of backbone flexibility, were excluded from the fitting procedure. This resulted in a data set of 140 experimental RDC values. Q values were calculated to assess the quality of the fits between experimental and calculated RDC values (Cornilescu et al. 1998).

## Extent of assignments and data deposition

The 2D ^1^H-^15^N BEST-TROSY spectrum of apo-KKT23^125–348^ shows fewer than the expected number of peaks (~ 170 peaks observed) for a protein that contains 216 non-proline residues (Supporting Fig. 1). The peaks in the spectrum are well dispersed, suggesting that the apo-protein is folded. The 2D ^1^H-^15^N BEST-TROSY spectrum of KKT23^125–348^ with an excess of the co-factor acetyl-CoA shows significant chemical shift changes compared to that of apo-KKT23^125–348^ (Supporting Fig. 1). Acetyl-CoA contains an aromatic adenine moiety and negatively-charged phosphate groups; many of the observed chemical shift differences may arise from aromatic ring current shifts, electrostatic and hydrogen bonding contributions to the shifts arising upon binding of acetyl-CoA. In the presence of acetyl-CoA, ~ 25 additional peaks were observed, leaving ~ 20 peaks missing; this might result from overlap, from rapidly exchanging amides or due to specific exchange broadening. Significant degradation of the apo-KKT23 was observed over a period of 24 h; this was not observed upon addition of acetyl-CoA. Based on the observations that acetyl-CoA stabilises KKT23^125–348^ and that the acetyltransferase domain in complex with acetyl-CoA is of more functional interest, further NMR studies were carried out on KKT23^125–348^ in the presence of acetyl-CoA.

**Fig. 1:**
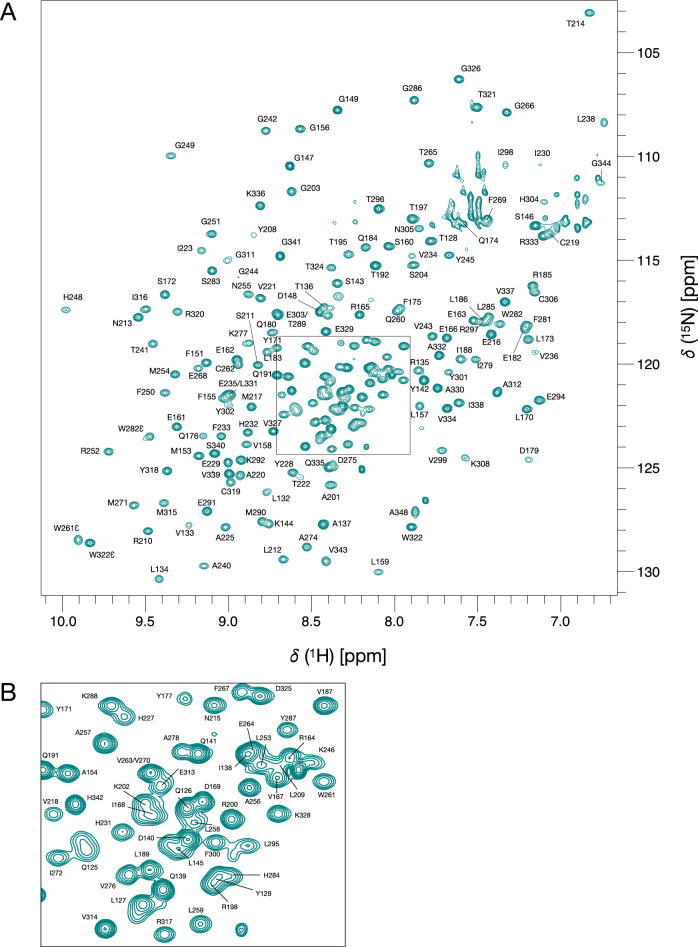
**A** 750 MHz ^1^H–^15^N BEST-TROSY spectrum of KKT23^125–348^ bound to acetyl-CoA. The peak assignments for backbone amides are annotated. Some peaks in the region of 111–114 ppm and upfield of ~ 7.7 ppm are artefacts in the BEST-TROSY arising from incomplete cancellation of signals from the side chain amides of Asn and Gln which have not been assigned. **B** Expansion of the crowded region highlighted with a black box in (**A**)

The ^1^H-^15^N BEST-TROSY spectrum of KKT23^125–348^ in the presence of acetyl-CoA shows approximately 200 peaks of uniform intensity and good chemical shift dispersion (Fig. 1). To obtain backbone and side chain assignments, triple resonance data were acquired and analysed at pH 7 and 20 °C. ^1^H^N^ and ^15^N backbone resonances for 195 of the 216 non-proline residues of KKT23^125–348^ were assigned; ^1^HN^N^-^15^N peaks for S130, Q131, L132, H193, S194, R196, R199, Y205, T206, M224, H226, K247, S273, S293, Y307, K308, F309, K310, L345, M346 and D347 were not identified. There are a small number of unassigned peaks visible in the BEST-TROSY spectrum but most of these did not give rise to unambiguous correlations in triple resonance spectra so could not be assigned on the basis of these spectra alone. Additional statistics for the extent of ^1^H, ^13^C and ^15^N assignments are presented in Table 1.

**Table 1:** Extent of assignment for KKT23^125–348^

Percent Assigned						
Sample^a^	^1^H^N^/^15^N^b^	^13^C’	^1^Hα/^13^Cα	^1^Hβ/^13^Cβ	^1^Hγ/^13^Cγ^c^	^1^Hγ/^13^Cδ^d^
KKT23^125–348^ with acetyl-CoA	91.2/91.2	89.7	76.5/96.0	50.7/81.9	37.3/45.6	23.5/22.3
KKT23^125–348^ with CoA^e^	91.2/91.2	84.4	–/92.9	–/46.7	–/–	–/–

^a^The protein contains an N-terminal Gly-Ser sequence, which remains after TEV protease cleavage, preceding the native KKT23 sequence; assignments for these residues were not carried out. Assignment statistics are reported for the 224 residues of the native sequence.

^b^Backbone ^15^N statistics do not include proline nitrogens.

^c^Gamma carbons from Asp, Asn, His, Phe, Tyr and Trp, which do not have attached ^1^H and are generally not assigned, are not included in the statistics.

^d^Delta carbons from Glu, Gln and Trp (δ2), which do not have attached ^1^H and are generally not assigned, are not included in the statistics.

^e^Fewer triple resonance experiments were carried out for KKT23^125–348^ with CoA so the assignments are limited to ^1^H^N^, ^15^N, ^13^C’, ^13^Cα and ^13^Cβ.

**Fig. 2:**
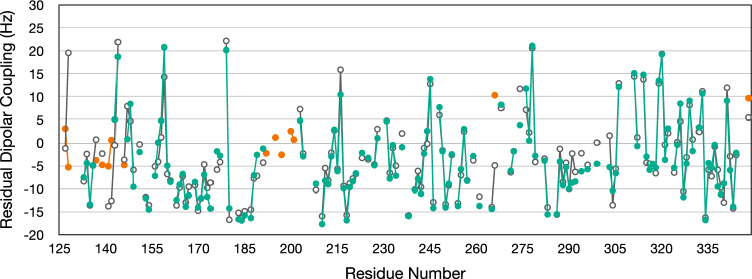
Experimental residual dipolar couplings (RDCs) (green circles) used in the fitting of the alignment tensor are plotted as a function of the sequence of KKT23^125–348^. Experimental residual dipolar couplings (RDCs) not used in the fitting procedure are shown as orange circles; these include residues 192, 195, 197, 200 and 201, for which no coordinates are available, and residues 127, 128, 137, 139, 141, 142, 146 and 348, which have low {^1^H}-^15^N heteronuclear NOE values. RDCs calculated using the 9EVQ X-ray structure are plotted for the 139 residues used in the fitting process and for the 8 residues with low heteronuclear NOE values (open circles) (see also Supporting Fig. 2)

RDC values ranging from − 17.6 Hz to + 21.0 Hz were measured for KKT23^125−348^ in 5% C12E6/hexanol (Fig. 2). Residues located in the loop region involving T192 – R200 have small experimental RDCs (− 2.6 to +2.5 Hz); this is consistent with the absence of electron density for these residues in the X-ray structure which suggests a mobile loop. The molecular alignment tensor in the X-ray structure was fitted to minimise the *χ*^2^ between the experimental and calculated RDCs for 140 residues. A Q value of 0.28 was obtained; one residue, G266, contributes significantly to this Q value with a difference between experimental and calculated RDCs of 14.6 Hz (Fig. 2). G266 is a surface exposed residue located in a loop between an α-helix and a β-strand and it is possible that the conformation of this residue differs in the crystal and in solution. If G266 is removed from the data set then the remaining 139 RDCs give a Q value of 0.24 with D_a_ and R values of 11.2 and 0.33, respectively (Fig. 2 and Supporting Fig. 2).

The fitted alignment tensor can be used to predict RDC values for all residues observed in the X-ray structure. These can be compared with the experimental values measured for some of the unassigned, but well-resolved, peaks in Fig. 1. Backbone ^1^H^N^/^15^N peaks at 8.77/126.2 ppm and 7.58/124.5 ppm can be assigned to L132 and K308 on the basis of the RDCs and weak correlations observed in triple-resonance spectra.

In addition, three tryptophan ^1^Hε_1_/^15^Nε_1_ peaks can be assigned on the basis of the RDC values and observed NOEs. W322 is the only tryptophan predicted to have a positive RDC value (+ 8.8 Hz) for Hε_1_; an RDC of + 9.0 Hz is measured for the peak at 9.84/128.7 ppm. W282 is predicted to have the smallest negative RDC value (− 6.6 Hz) for Hε_1_; an RDC of − 4.0 Hz is measured for the peak at 9.48/123.5 ppm; in addition, this Hε_1_ has an NOE to I272 Hγ_2_ as predicted from the X-ray structure. The ^1^Hε_1_/^15^Nε_1_ peak at 9.90/128.4 ppm is assigned to W261 on the basis of similar experimental and predicted RDCs (− 9.2 Hz and − 8.2 Hz, respectively) and an NOE to T265 Hγ predicted from the X-ray structure.

In the catalytic cycle of an acetyltransferase, the acetyl group of acetyl-CoA is transferred to substrate leaving the enzyme bound to CoA. We have also obtained backbone assignments for KKT23^125–348^ in the presence of CoA. ^1^H^N^ and ^15^N backbone resonances for 197 of the 216 non-proline residues of KKT23^125–348^ were assigned. The same group of 19 residues not assigned in the presence of acetyl-CoA were also not assigned in the spectrum with CoA. Further statistics for the extent of ^1^H, ^13^C and ^15^N assignments are presented in Table 1.

**Fig. 3:**
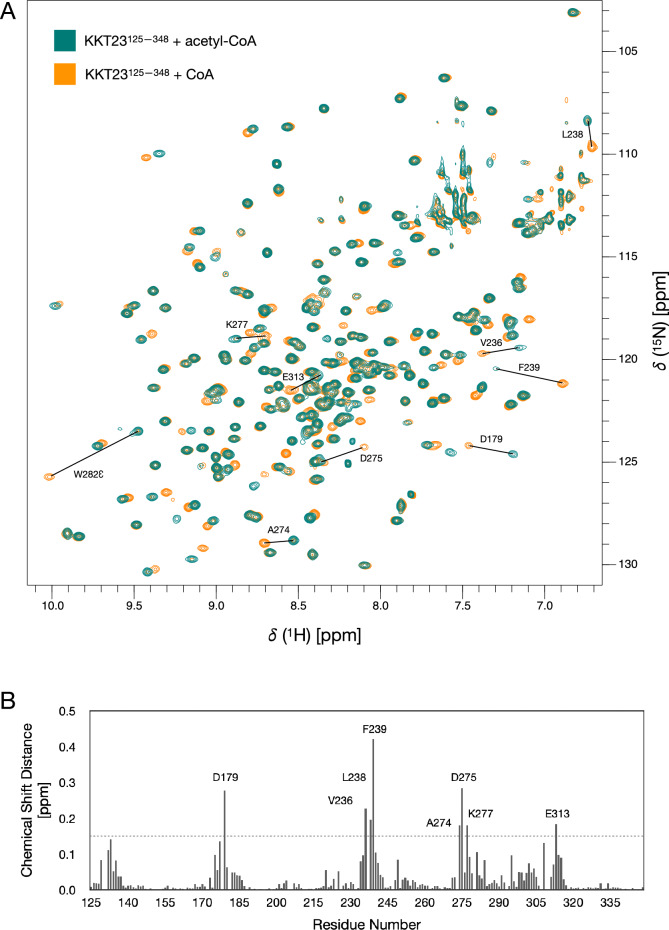
**A** Overlay of 750 MHz ^1^H–^15^N BEST-TROSY spectra of KKT23^125–348^ bound to acetyl-CoA (teal) and to CoA (orange). Peaks corresponding to residues with large chemical shift differences (> 0.15 ppm) between the acetyl-CoA and CoA bound states are labelled and a black line connects the peaks from the two states. **B** Combined chemical shift changes observed between peak positions in the presence of acetyl-CoA and CoA. The combined chemical shift changes were calculated using ((Δ^1^H^N^)^2^ + (0.15 × Δ^15^N)^2^)^1/2^. Residues with shift changes of > 0.15 ppm are indicated by the dashed line and are labelled in both panels

The overlay of the spectra of KKT23^125–348^ in the presence of acetyl-CoA and CoA (Fig. 3A) shows that the majority of peaks do not shift as a result of the loss of the acetyl group (Fig. 3B). However, larger combined chemical shift changes, of greater than 0.15 ppm, are observed for 8 residues. In addition, the side chain ^1^Hε_1_/^15^Nε_1_ peak of W282 has a combined chemical shift change of 0.66 ppm. L238, F239, D275 and W282 have backbone and/or side chain atoms within 5 Å of the acetyl group in the X-ray structure of KKT23^125–348^ bound to acetyl-CoA. Creation of a larger pocket upon loss of the acetyl group is likely to lead to small structural rearrangements in this region. The observed chemical shift changes may be a direct result of a change in position of a residue or could be the indirect result of changes in ring current effects due to movements of F239 and W282.

The ^1^H, ^13^C and ^15^N chemical shift assignments presented here for KKT23^125–348^ bound to acetyl-CoA or CoA have been deposited in the BioMagResBank (https://bmrb.io/) under the accession numbers 52461 and 52465, respectively. These assignments provide the starting point in detailed investigation of the KKT23 structure, dynamics and interactions in solution.

## Supplementary Information

Below is the link to the electronic supplementary material.

Supplementary (PDF 498 KB)

## Data Availability

Assignments for the KKT23 C-terminal domain have been deposited in the BMRB (https://bmrb.io) under accession numbers 52461 (KKT23 bound to acetyl-CoA) and 52465 (KKT23 bound to CoA). The plasmids used in this study are available upon request.
