# Supplementary material for: 1H, 13C and 15N resonance assignments for the acetyltransferase domain of the kinetoplastid kinetochore protein KKT23 from Trypanosoma brucei
Source: Biomol NMR Assign. 2025 May 2;19(1):187–94. doi: 10.1007/s12104-025-10235-4 (PMC12116700; doi:10.1007/s12104-025-10235-4)
Supplement: Supplementary file 1 — Supplementary (PDF 498 KB) [file 12104_2025_10235_MOESM1_ESM.pdf]

SUPPORTING INFORMATION FOR:

^{1}H, ^{13}C and ^{15}N resonance assignments for the acetyltransferase domain of the kinetoplastid kinetochore protein KKT23 from *Trypanosoma brucei*

Patryk Ludzia[1,2], Charlotte Nugent[1], Bungo Akiyoshi[1,3] and Christina Redfield[1*]*

[1]Department of Biochemistry, University of Oxford, Oxford, OX1 3QU, UK

[2]Department of Biochemistry, University of Cambridge, Cambridge, CB2 1GA, UK

[3]Institute of Cell Biology, School of Biological Sciences, University of Edinburgh, Edinburgh, EH9 3BF, UK

[*]Corresponding authors:

B. Akiyoshi bungo.akiyoshi@ed.ac.uk Tel: +44 (0) 1316 50 7005

C. Redfield christina.redfield@bioch.ox.ac.uk Tel: +44 (0)1865 275330

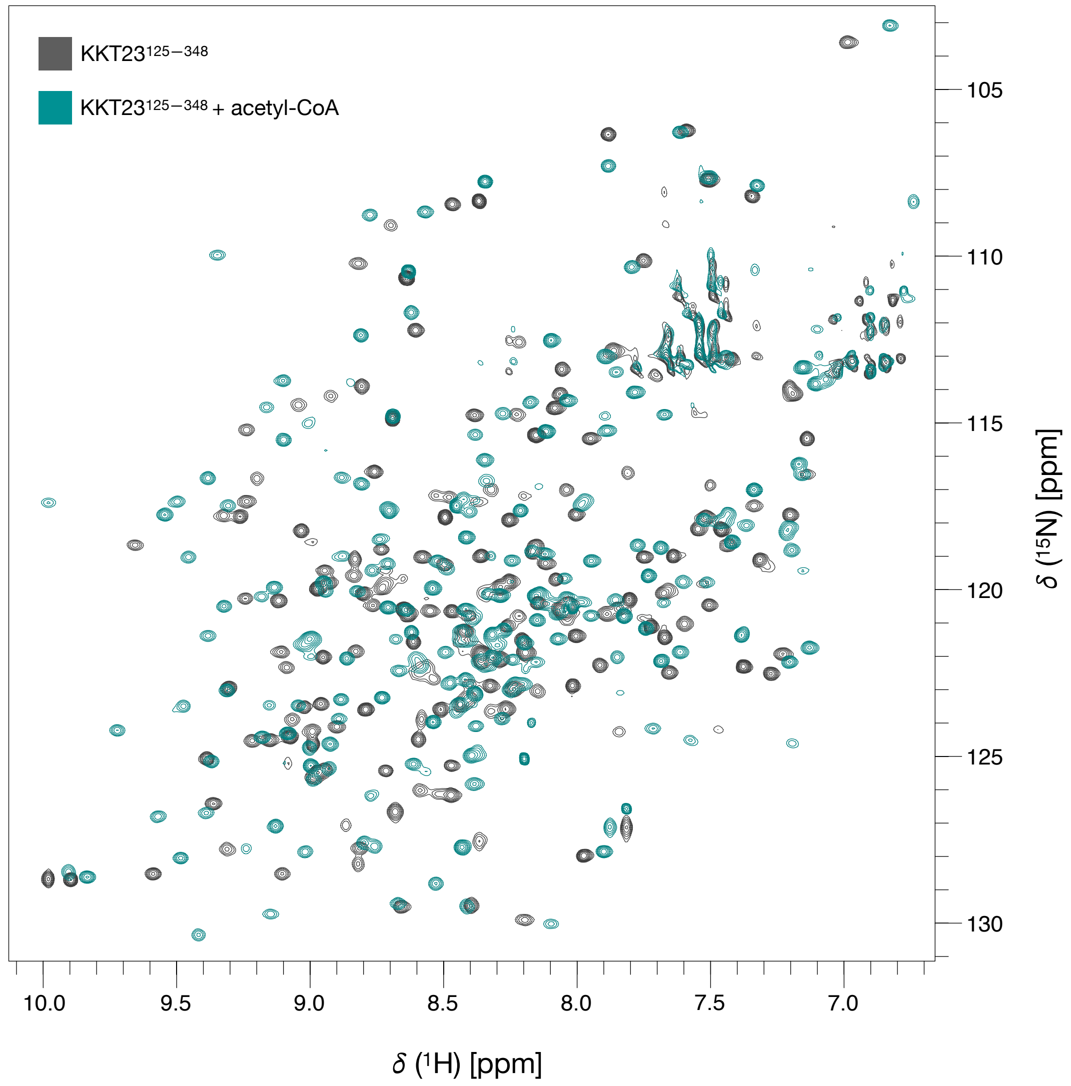

Supporting Figure 1

Overlay of 750 MHz ^{1}H–^{15}N BEST-TROSY spectra of apo-KKT23$^{125–238}$ (grey) and KKT23$^{125–238}$ bound to acetyl-CoA (teal). Some peaks in the region of 111–114 ppm and upfield of ~7.7 ppm are artefacts in the BEST-TROSY arising from incomplete cancellation of signals from the side chain amides of Asn and Gln.

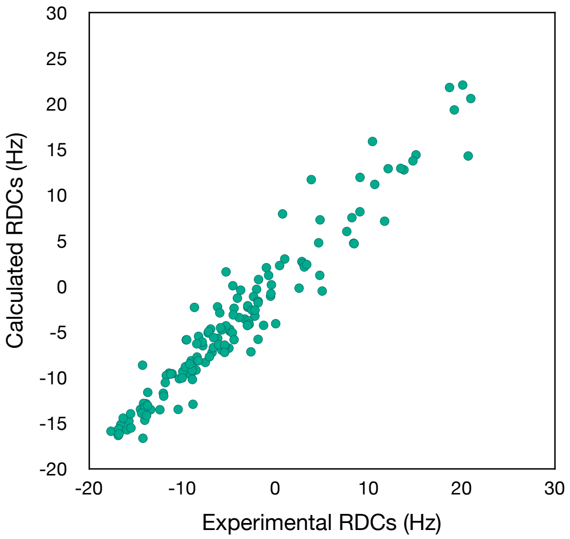

Supporting Figure 2

Plot of the experimental RDCs versus the calculated RDCs. The good agreement between experimental and calculated RDCs is shown by the Q value of 0.24.
